# A Dual-Targeting Peptide Inhibitor Simultaneously Blocking Viral Attachment and Membrane Fusion for Broad-Spectrum Inhibition of SARS-CoV-2

**DOI:** 10.3390/ijms26125729

**Published:** 2025-06-15

**Authors:** Wenwen Bi, Tao Zhu, Yawen Xu, Jianmin Li

**Affiliations:** 1 Laboratory of Advanced Biotechnology, ZJU-Hangzhou Global Scientific and Technological Innovation Center, Zhejiang University, Hangzhou 311215, China; l210892@zju.edu.cn (T.Z.); l241261@zju.edu.cn (Y.X.); 2 Laboratory of Advanced Biotechnology, Beijing Institute of Biotechnology, Beijing 100071, China

**Keywords:** SARS-CoV-2, dual-targeting peptide inhibitor, RBD, HR1, broad-spectrum antiviral

## Abstract

The emergence of severe acute respiratory syndrome coronavirus 2 (SARS-CoV-2) variants with enhanced transmissibility and immune evasion underscores the urgent need for broad-spectrum antiviral therapeutics. In this study, we strategically engineered a novel dual-targeting peptide inhibitor, R1L25HR2, by conjugating the receptor-binding domain (RBD)-targeting peptide R1 with the heptad repeat 1 (HR1)-targeting peptide HR2 through an optimized 25-mer flexible linker (GGGGS)5, aiming to simultaneously block viral attachment and membrane fusion. R1L25HR2 potently and broadly inhibits the infection of SARS-CoV-2 and its emerging variants, including recent circulating strains JN.1 and KP.2, with IC_50_ values ranging from 5.3 to 253.5 nM, which is significantly more effective than HR2 and R1 alone. Mechanistically, R1L25HR2 inhibits viral attachment and membrane fusion by binding to both RBD and HR1 with low nanomolar affinity. These results highlight the innovative strategy of dual-targeting the RBD and HR1 domains as an effective approach to overcome viral resistance and achieve broad-spectrum antiviral activity.

## 1. Introduction

At the end of 2019, a novel coronavirus, namely severe acute respiratory syndrome coronavirus 2 (SARS-CoV-2), emerged and spread rapidly the worldwide. As of 18 May 2025, the World Health Organization (WHO) has reported over 777 million confirmed cases of coronavirus disease 2019 (COVID-19) and approximately 7.1 million deaths globally (https://data.who.int/dashboards/covid19/cases (accessed on 7 June 2025)). This pandemic has posed unprecedented challenges to global public health and economic stability. Despite the rapid development of vaccines and antiviral drugs, the emergence of SARS-CoV-2 variants, particularly the Omicron strain and its sublineages, has significantly impaired the clinical efficacy of many vaccines and antibody therapies [1,2,3,4]. This has led to increased rates of breakthrough infections and reinfections, highlighting the critical need for broad-spectrum antiviral therapeutics effective against current and future variants of SARS-CoV-2.

The spike (S) protein of SARS-CoV-2 plays a pivotal role in viral entry by mediating the attachment of the virus to the host cell angiotensin-converting enzyme 2 (ACE2) receptor through its receptor-binding domain (RBD) and subsequent membrane fusion facilitated by the heptad repeat 1 (HR1) and heptad repeat 2 (HR2) regions [5,6,7,8,9,10]. Thus, disrupting either the RBD/ACE2 binding or HR1/HR2-mediated fusion presents promising antiviral strategies. While peptide inhibitors targeting either the RBD or the HR1 region have been identified [11,12,13,14,15], single-target peptides often exhibit limited efficacy or vulnerability to viral escape mutations. For instance, the computationally de novo designed LCB1 peptide (56 amino acids) exhibits potent SARS-CoV-2 inhibition through high-affinity binding to RBD [11]. However, its efficacy is significantly diminished against emerging variants, as evidenced by reduced binding affinity and antiviral activity [16,17]. Similarly, phage display screening has identified the RBD-binding R1 peptide, which demonstrates high binding affinity but lacks antiviral activity [13]. In contrast, unmodified HR1-targeting peptides demonstrate antiviral effects but are less effective against emerging variants [14,18].

Dual-targeting strategies that simultaneously block viral attachment and fusion may overcome these limitations by requiring concurrent mutations in both RBD and HR1 for viral escape. Our previous development of A1L35HR2m, an ACE2-HR2 fusion peptide with pan-coronavirus activity but limited RBD binding affinity [19], led us to hypothesize that coupling RBD- and HR1-targeting peptides could synergistically block viral entry at both attachment and fusion stages.

Here, we designed a dual-targeting peptide inhibitor, R1L25HR2, by fusing the R1 peptide with the HR2-derived peptide via a flexible linker. This innovative strategy aims to simultaneously block viral attachment and membrane fusion, thereby enhancing antiviral potency and breadth. Our bio-layer interferometry (BLI) assays confirmed that R1L25HR2 binds to both the RBD and HR1 with low nanomolar affinity, indicating its dual-targeting capability. Moreover, pseudovirus inhibition assays demonstrated that R1L25HR2 exhibits significantly enhanced antiviral activity compared to the HR2 peptide alone. Remarkably, R1L25HR2 displays broad-spectrum efficacy against SARS-CoV-2 and its emerging variants, including JN.1 and KP.2, with inhibitory concentrations in the nanomolar range.

## 2. Results

### 2.1. Design and Identification of the Bispecific Peptide Inhibitors

**Figure 1:**
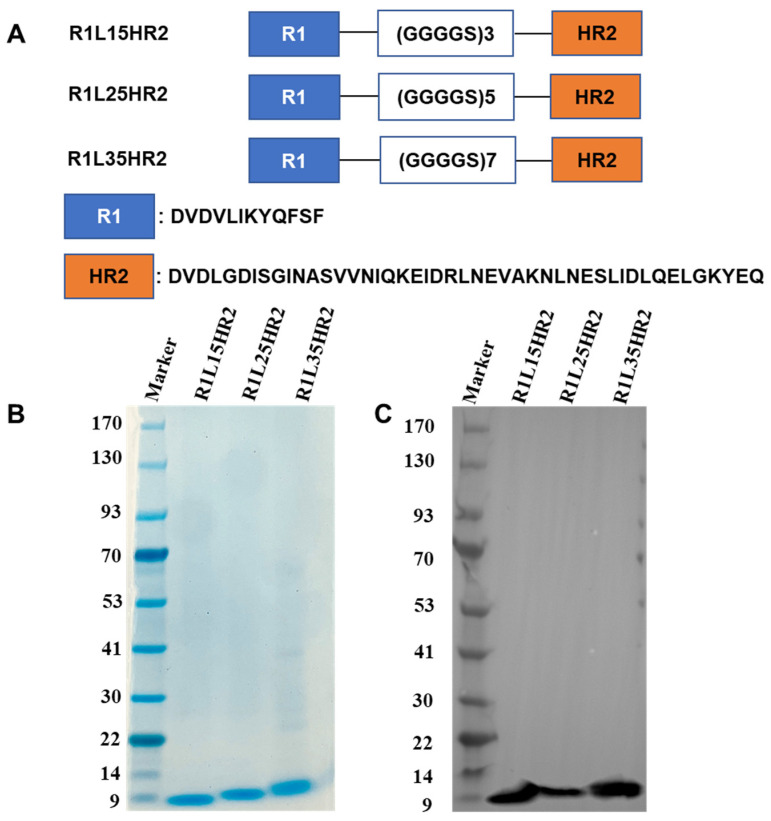
Design and identification of R1L15HR2, R1L25HR2 and R1L35HR2. Their design (**A**), identification by SDS-PAGE (**B**), and confirmation via Western blot (**C**).

To develop a potent bispecific SARS-CoV-2 entry inhibitor, the RBD-targeting peptide R1 was genetically fused to the N-terminus of the HR1-targeting peptide HR2 using flexible linkers (GGGGS)n. Three constructs were generated with distinct linker lengths: (GGGGS)3, (GGGGS)5 and (GGGGS)7, designated as R1L15HR2, R1L25HR2 and R1L35HR2, respectively (Figure 1A). All fusion peptides were recombinantly expressed in *E. coli* and purified via Ni-NTA beads. Purity and molecular weight were verified by SDS-PAGE (Figure 1B) and Western blotting (Figure 1C) under reducing conditions.

### 2.2. Conjugating RBD- and HR1-Targeting Peptides Could Synergistically Inhibit SARS-CoV-2 Infection

**Figure 2:**
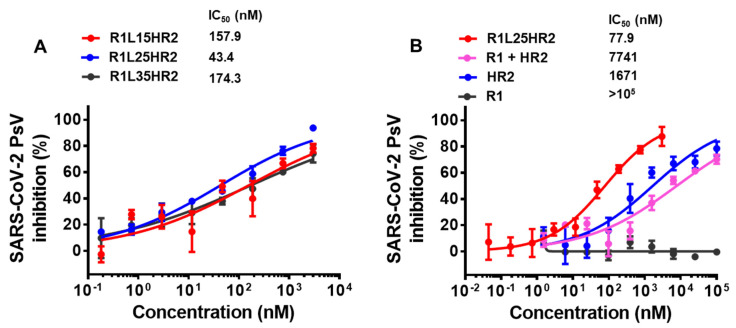
Conjugating the RBD-targeting peptide R1 to the N-terminus of the HR2 peptide significantly enhanced the inhibitory activity against SARS-CoV-2 infection. (**A**) Inhibitory activity of R1L15HR2, R1L25HR2 and R1L35HR2 against SARS-CoV-2 pseudovirus infection in Caco-2 cells. (**B**) Inhibitory activity of R1L25HR2, HR2, R1 and R1/HR2 mixtures against SARS-CoV-2 pseudovirus infection in Caco-2 cells. Each sample was tested in triplicate and the data are presented as the mean ± SEM.

To evaluate the inhibitory activities of R1L15HR2, R1L25HR2 and R1L35HR2, we conducted a SARS-CoV-2 pseudotyped virus infection assay as previously described [19,20]. Our results revealed that R1L25HR2 exhibited remarkable antiviral activity with an IC_50_ value of 43 nM, approximately 4-fold more potent than R1L15HR2 (IC_50_ = 158 nM) and R1L35HR2 (IC_50_ = 174 nM) (Figure 2A). This indicates the crucial role of linker length in optimizing spatial coordination for dual-targeting synergy. Furthermore, R1L25HR2 demonstrated a 99-fold greater potency (IC_50_ = 78 nM) compared to the mixture of R1/HR2 peptides (IC_50_ = 7741 nM) in pseudovirus inhibition assays (Figure 2B). These findings conclusively demonstrate that covalent conjugation of RBD-targeting R1 to HR2 through an optimized flexible linker creates a bifunctional inhibitor with cooperative effects, significantly outperforming monovalent agents or their unlinked combination.

### 2.3. R1L25HR2 Inhibited SARS-CoV-2 Entry at the Early Stage and Exhibited a High Affinity to RBD and HR1

**Figure 3:**
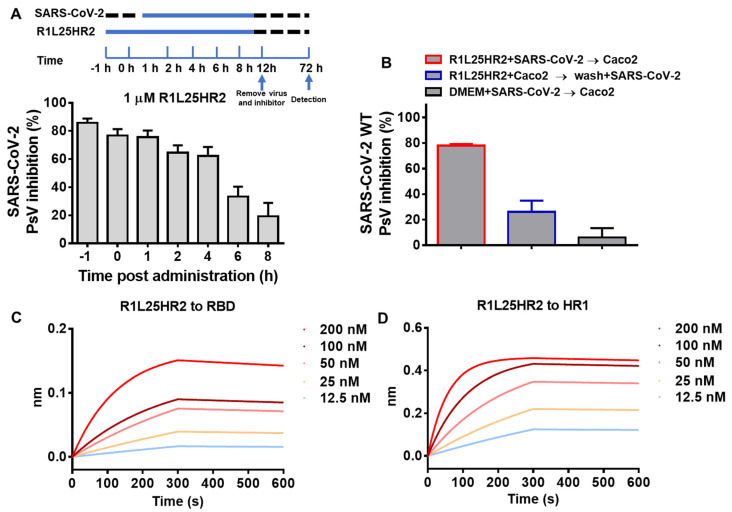
R1L25HR2 inhibits SARS-CoV-2 pseudovirus entry at the early stage and its binding affinity to RBD and HR1. (**A**) The upper scheme diagram shows the procedure for the time-of-addition assay. The inhibitory activity of 1 μM R1L25HR2 against SARS-CoV-2 pseudovirus infection at different intervals post-infection. (**B**) A washout assay was performed to determine whether R1L25HR2 acts on the virus or cells. (**C**) Binding affinity of R1L25HR2 to RBD was determined by BLI. (**D**) Binding affinity of R1L25HR2 to HR1 was determined by BLI. The fitting curves were analyzed by ForteBio Data Analysis 12.0 software. Each sample was tested in triplicate and the data are presented as the mean ± SEM.

To explore the stage of SARS-CoV-2 entry targeted by R1L25HR2, a time-of-addition assay was performed. As shown in Figure 3A, when 1 μM R1L25HR2 was added to Caco-2 cells within 1 h post-SARS-CoV-2 pseudovirus infection, it maintained roughly 80% inhibitory efficacy. However, with the delay of the addition time, the entry inhibitory activity of R1L25HR2 gradually diminished and dropped to 20% when R1L25HR2 was added 8 h later. This result indicates that R1L25HR2 predominantly exerts its inhibitory effect during the early phase of SARS-CoV-2 pseudovirus entry.

To investigate the mechanism by which R1L25HR2 inhibits SARS-CoV-2 entry, we conducted two binding assays focusing on the host cell factors and viral proteins. In the washout assay, Caco-2 cells were incubated with R1L25HR2 at 37 °C for 30 min, then washed with DMEM before adding SARS-CoV-2 pseudovirus. As shown in Figure 3B, R1L25HR2 preserved about 80% inhibitory efficacy when the cells were not washed (column 1), but almost lost its inhibitory activity when washed before pseudovirus infection (column 2). This result indicates that the inhibition of viral entry by R1L25HR2 is not dependent on interacting with host cell factors. Next, we used the RBD and HR1 peptide derived from SARS-CoV-2 S protein to evaluate the binding affinity of R1L25HR2. As expected, R1L25HR2 could bind to the RBD and HR1 with dissociation constants (K_D_) of 5.02 and 0.85 nM, respectively (Figure 3C,D and Table 1). These results suggest that R1L25HR2 inhibits SARS-CoV-2 pseudovirus entry through direct interaction with the RBD and HR1 of S protein.

### 2.4. R1L25HR2 Inhibited SARS-CoV-2 Infection by Preventing Cell Surface Attachment and Blocking S Protein-Mediated Membrane Fusion

**Figure 4:**
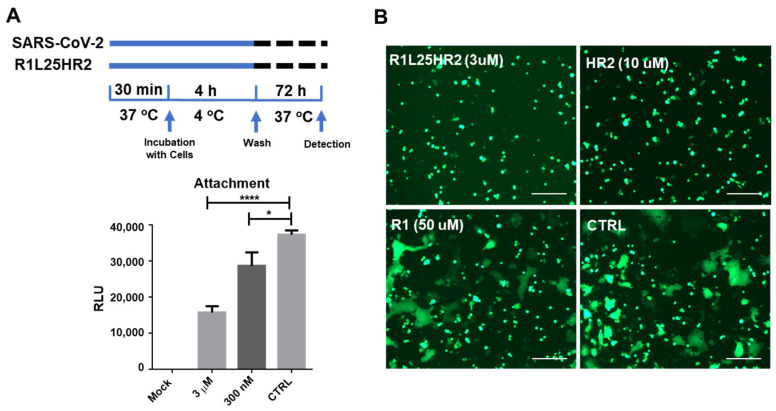
R1L25HR2 inhibits SARS-CoV-2 infection by preventing cell surface attachment and blocking S protein-mediated membrane fusion. (**A**) The upper scheme diagram illustrates the procedure for the attachment assay. The luciferase luminescence of R1L25HR2-treated cells was analyzed to determine whether R1L25HR2 can block the attachment of SARS-CoV-2. (**B**) A SARS-CoV-2 S protein-mediated cell–cell fusion assay was performed to determine whether R1L25HR2 can inhibit S protein-mediated membrane fusion. Scale bars = 100 μm. Each sample was tested in triplicate and the data are presented as the mean ± SEM. An unpaired non-parametric Mann–Whitney test was performed. * *p* < 0.05 and **** *p* < 0.0001.

It has been demonstrated that SARS-CoV-2 S-mediated viral entry consists of at least five steps, including cell surface attachment, receptor binding, conformational transition, transmembrane serine protease 2 (TMPRSS2) or cathepsin L (CTSL) cleavage of S2 protein, and membrane fusion [21,22,23,24,25,26]. The results of the time-of-addition assay in Figure 3A show that R1L25HR2 inhibits the entry of SARS-CoV-2 at an early step. To determine whether R1L25HR2 can block the viral attachment to target cells, the attachment assay was performed. At 4 °C, actin polymerization and depolymerization slow significantly compared to the physiological temperature, yet the interaction between virions and host factors on the cell surface remains unaffected [27]. Thus, we incubated SARS-CoV-2 pseudovirus with Caco-2 cells at 4 °C to allow SARS-CoV-2 pseudovirus binding to the cell surface, then washed away unbound SARS-CoV-2 before shifting the cells to 37 °C for further incubation. As shown in Figure 4A, R1L25HR2-treated cells exhibited significantly reduced luciferase luminescence compared to untreated cells, indicating that R1L25HR2 blocks SARS-CoV-2 entry during the attachment phase.

To further investigate whether R1L25HR2 can inhibit the fusion between SARS-CoV-2 and target cell membranes, we employed the S protein-mediated cell–cell fusion assay as previously described [18,20]. Our results showed that R1L25HR2 and HR2 peptides completely inhibited SARS-CoV-2 D614G S protein-mediated cell–cell fusion between 293T/EGFP/S and Caco-2 cells at concentrations of 3 μM and 10 μM, respectively. In contrast, even at concentrations up to 50 μM, the R1 peptide was unable to inhibit this fusion process (Figure 4B). This result indicates that R1L25HR2 inhibits the fusion between viral and target cell membranes.

### 2.5. R1L25HR2 Exhibited Broad and Potent Inhibitory Activity Against Different SARS-CoV-2 Variants

**Figure 5:**
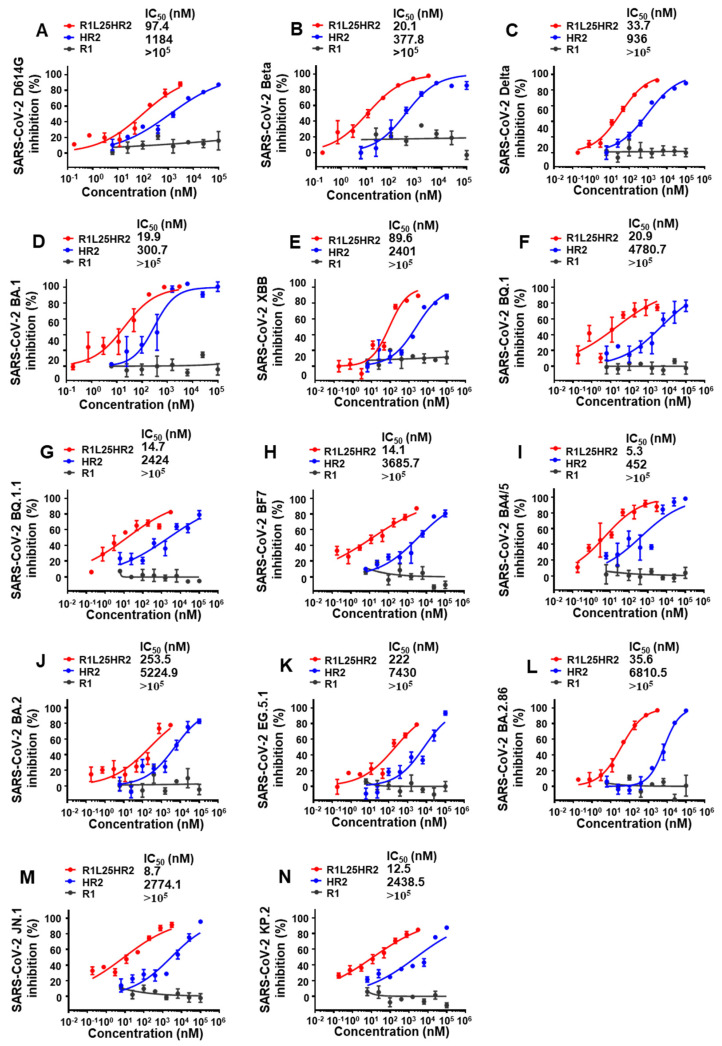
R1L25HR2 broadly and potently inhibited diverse SARS-CoV-2 variants in pseudovirus infection assays. Inhibitory activity of R1L25HR2, HR2 and R1 in pseudovirus infection assays against SARS-CoV-2 D614G (**A**), Beta (**B**), Delta (**C**), BA.1 (**D**), XBB (**E**), BQ.1 (**F**), BQ.1.1 (**G**), BF7 (**H**), BA4/5 (**I**), BA.2 (**J**), EG.5.1 (**K**), BA.2.86 (**L**), JN.1 (**M**) and KP.2 (**N**). Each sample was tested in triplicate and the data are presented as the mean ± SEM.

To evaluate the inhibitory effects of R1L25HR2, HR2 and R1 peptides on SARS-CoV-2 variants, we conducted comprehensive infection inhibition assays with different SARS-CoV-2 pseudovirus strains including D614G, Beta, Delta and multiple Omicron sublineages (BA.1, XBB, BQ.1, BQ.1.1, BF7, BA4/5, BA.2, EG.5.1, BA.2.86, JN.1 and KP.2). R1L25HR2 exhibited significantly more potent inhibitory activity against all tested SARS-CoV-2 variants with IC_50_ values ranging from 5.3 to 253.5 nM. In contrast, the HR2 peptide exhibited lower inhibitory efficacy, with IC_50_ values ranging from 0.3 to 7.4 μM (Figure 5A–N). Notably, R1L25HR2 maintained its inhibitory potency across all variants, highlighting its broad-spectrum antiviral activity. These results underscore the superior performance of R1L25HR2 in inhibiting SARS-CoV-2 entry compared to HR2 and R1 peptides.

### 2.6. R1L25HR2 Showed No Cytotoxicity to Target Cells

**Figure 6:**
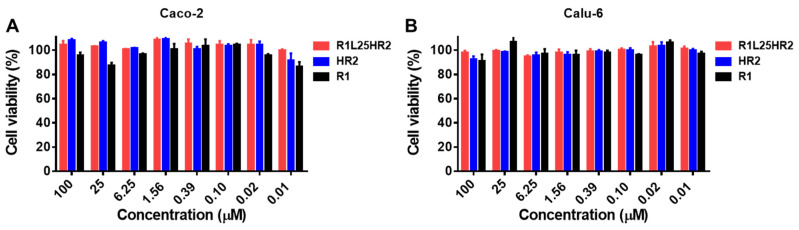
The cytotoxicity of R1L25HR2 to Caco-2 and Calu-6 cells. The effect of R1L25HR2, HR2 and R1 on the viability of Caco-2 (**A**) and Calu-6 (**B**) cells.

The cytotoxic effect of R1L25HR2 was evaluated in Caco-2 and Calu-6 cells using the Cell Counting Kit-8 (CCK-8) assay. Cells were treated with a concentration gradient of R1L25HR2, HR2 and R1 for 48 h under standard culture conditions. As shown in Figure 6A,B, treatment with R1L25HR2 at concentrations up to 100 μM maintained 90% cell viability in both cell lines. This maximal non-toxic concentration represents a 2325-fold excess over the peptide’s half-maximal inhibitory concentration (IC_50_) against SARS-CoV-2 infection, demonstrating a wide safe therapeutic window.

## 3. Discussion

The rapid evolution of SARS-CoV-2 variants, such as JN.1 and KP.2, highlights the critical need for therapeutic strategies that address viral evolution through multi-mechanistic action. Our development of R1L25HR2, a dual-targeting peptide inhibitor that targets the RBD and HR1 of the spike protein, demonstrates the efficacy of rational design in overcoming these challenges. By blocking viral attachment via RBD binding and membrane fusion via HR1 interaction, R1L25HR2 achieves broad-spectrum efficacy against diverse variants, with IC_50_ values ranging from 5.3 to 253.5 nM, significantly outperforming its individual components (R1 or HR2 alone). These findings are consistent with the success of bispecific inhibitors like 212-148 and bivalent proteins such as GL25E [28,29], which have demonstrated that dual-targeting can create a higher genetic barrier against viral escape.

To combat viral escape and resistance, we developed bispecific peptides targeting the RBD and HR1 of the S protein. Given the ~100 Å separation between these domains in the prefusion conformation, we used a rational linker design with flexible (GGGGS)n repeats of varying lengths (L15, L25 and L35) to enable dual domain engagement. The high flexibility of the GGGGS linker minimizes steric hindrance between the two functional domains, allowing R1 and HR2 to independently interact with their respective targets on the SARS-CoV-2 spike protein. Previous studies in peptide and protein engineering have demonstrated that GGGGS linkers effectively preserve the bioactivity of fused peptides and proteins [19,29,30], which guided our choice of the GGGGS linker for R1 and HR2 peptides’ fusion. Our results indicated that R1L25HR2, using a flexible 25-mer linker, had the strongest antiviral activity, even though R1 cannot inhibit SARS-CoV-2 infection and HR2 has lower activity. The flexible linker likely ensures R1/RBD and HR2/HR1 interactions, effectively inhibiting viral infection. Consistent with this mechanism, BLI results confirmed R1L25HR2’s dual high-affinity binding, with dissociation constants of 5.02 nM for RBD and 0.85 nM for HR1.

Our results demonstrated that R1L25HR2 inhibited SARS-CoV-2 infection by blocking cell surface attachment and S protein-mediated membrane fusion (Figure 4). Specifically, the R1 of R1L25HR2 interacts with the RBD, trapping the spike protein in its prefusion state and thereby preventing viral attachment. Subsequently, HR2 binds to HR1, blocking the fusion of the cell and viral membranes. This dual-targeting mechanism aligns with recent cryo-EM studies of similar dual-functional proteins, such as AL5E [30], which demonstrate that simultaneous engagement of both RBD and HR1 can significantly enhance the inhibitory effect on viral entry.

Importantly, R1L25HR2 maintains its potency against highly mutated variants like JN.1 (IC_50_ = 8.7 nM) and KP.2 (IC_50_ = 12.5 nM). Its resistance to antigenic drift is likely due to the dual-targeting approach, which reduces the likelihood of escape mutations arising simultaneously in both the RBD and HR1 domains. This strategy aligns with the increasing evidence that highlights the effectiveness of multi-target therapies in reducing viral resistance. Similar approaches have been successfully employed in other viral infections, such as the use of combination antiretroviral therapies for HIV [31]. R1L25HR2 not only enhances inhibitory potency but also poses a higher genetic barrier for the virus to escape by simultaneously targeting two critical and functionally distinct regions of the spike protein. Our findings are consistent with previous studies that have demonstrated the importance of dual targeting in reducing the likelihood of viral escape. For example, studies on bispecific antibodies targeting SARS-CoV-2 have shown that simultaneous engagement of multiple epitopes can significantly reduce the emergence of escape mutations [32,33,34]. Similarly, the development of dual-epitope peptides targeting different regions of the spike protein has been shown to enhance the breadth and potency of antiviral activity [19].

In conclusion, we have designed and characterized a dual-targeting peptide that simultaneously engages both RBD and HR1 domains of the SARS-CoV-2 spike protein. This rationally engineered chimeric peptide demonstrates potent and broad-spectrum inhibitory activity against SARS-CoV-2 and its emerging variants, overcoming the inherent limitations of single-target antiviral agents (R1 and HR2 peptides alone) and presenting a promising strategy for developing broad-spectrum therapeutics. However, several important questions remain for future investigation: (1) in vivo efficacy evaluation in animal models; (2) pharmacokinetic optimization through strategies like IBP fusion to extend half-life [35]; (3) exploration of potential synergistic effects with existing antivirals [36]; and (4) structural characterization of the peptide–spike protein interactions to guide further engineering. These studies will be crucial for advancing this dual-targeting strategy toward clinical application.

## 4. Materials and Methods

### 4.1. Plasmids and Cells

The envelope-expressing plasmid pcDNA3.1-SARS-CoV-2-S and the luciferase-expressing plasmid pNL4-3.Luc.RE were generously provided by Drs. Shibo Jiang and Lu Lu at Shanghai Medical College of Fudan University. The SARS-CoV-2 S mutant envelope-expressing plasmids were constructed via directed mutagenesis using overlap PCR. The pcDNA3.1 plasmid encoding EGFP fused with the SARS-CoV-2 D614G S protein or EGFP alone was stored in our laboratory. The 293T, Caco-2 and Calu-6 cell lines were cultured in Dulbecco’s Modified Eagle Medium (DMEM) supplemented with 10% fetal bovine serum (FBS).

### 4.2. Construction, Expression and Purification of Bispecific Peptides

The genes encoding R1L15HR2, R1L25HR2 and R1L35HR2 were amplified using overlap PCR and subsequently cloned into the pET28a vector. The resulting plasmids, pET28a-R1L15HR2, pET28a-R1L25HR2 and pET28a-R1L35HR2, were individually transformed into *E. coli* BL21 (DE3) competent cells. For protein expression, transformed cells were cultured in LB medium at 37 °C until the OD600 reached 0.6. Protein expression was induced with 0.5 mM isopropyl 1-thio-β-D-galactopyranoside (IPTG), and the cultures were incubated for an additional 3 h at 37 °C. Following induction, cells were harvested by centrifugation, resuspended in PBS containing 1% Triton X-100 and lysed by sonication. The lysate was clarified by centrifugation at 12,000× *g* for 10 min, and the supernatant was loaded onto Ni-NTA affinity beads (Smart-Lifesciences, Changzhou, China). The beads were washed with phosphate buffered saline (PBS) containing 10 mM imidazole, and the target proteins were eluted using elution buffer (250 mM imidazole in PBS).

### 4.3. Sodium Dodecyl Sulfate-Polyacrylamide Gel Electrophoresis (SDS-PAGE) and Western Blot Analysis

The purity and molecular weight of purified recombinant peptides were characterized by SDS-PAGE. Peptide samples were loaded onto 4–20% gradient SDS-PAGE and stained with Coomassie brilliant blue R-250. For Western blot analysis, the peptides were separated on a 4–20% gradient SDS-PAGE gel and then transferred to a polyvinylidene fluoride (PVDF) membrane. The membrane was blocked with a 5% (*w*/*v*) nonfat dry milk in Tris-buffered saline-Tween 20 (TBST, pH 7.4) for 1 h at room temperature. It was then incubated with horseradish peroxidase (HRP)-conjugated anti-His mouse monoclonal antibody (Zenbio, Chengdu, China) at a 1:5000 dilution for 1 h at room temperature. Following three washes with TBST, the membrane was incubated with an enhanced chemiluminescence (ECL) substrate (Biosharp, Hefei, China) and subsequently visualized using the ChemiScope 6100 chemiluminescence imaging system (Clinx Science Instruments, Shanghai, China).

### 4.4. Inhibition of Pseudotyped Virus Infection

SARS-CoV-2 pseudovirus and its variants were generated as previously described [19,37]. Briefly, 293T cells were co-transfected with a plasmid encoding the SARS-CoV-2 S protein (or its variants) and a luciferase-expressing HIV-1 genomic plasmid using Lipo2000 transfection reagent (Lablead, Beijing, China). Supernatants were harvested at 48 hrs post-transfection, centrifuged at 3000× *g* for 10 min and stored at −80 °C for single-cycle infection assays. For antiviral activity assessment, Caco-2 cells (10^4^/well) were infected with SARS-CoV-2 pseudovirus pre-incubated with inhibitors. After 16 h, the supernatants were replaced with fresh DMEM with 10% FBS, and cells were further cultured for 48 h at 37 °C. Luciferase activity was quantified by using a luciferase kit (Promega, Madison, WI, USA) on a Variokan LUX Multimode Microplate Reader (Thermo Fisher Scientific, Waltham, MA, USA-) following cell lysis. Dose–response inhibition curves were generated and analyzed using GraphPad Prism software (Version 6.02).

### 4.5. Binding Affinity Determination Using Bio-Layer Interferometry (BLI)

R1L25HR2 peptide was conjugated with biotin at a theoretical molar ratio of 1:3 using EZ-Link NHS-PEG12-Biotin (Thermo Fisher Scientific, Waltham, MA, USA) following the manufacturer’s instructions. Unreacted biotin was removed via ultrafiltration using a 3 KDa MWCO Amicon Ultra centrifugal filter (Merck Millipore, Darmstadt, Germany). For kinetic characterization, 5 µg/mL biotinylated R1L25HR2 was immobilized on streptavidin-coated biosensors. RBD and HR1 were serially diluted 1:2 starting at 200 nM with dilution buffer (PBS with 0.02% Tween 20). Reference sensors loaded with R1L25HR2 but not exposed to RBD or HR1 were used to measure and subtract background signals. Binding kinetics were analyzed using the ForteBio Data Analysis Software. Association rate constants (k_on_), dissociation rate constants (k_off_), and equilibrium dissociation constants (K_D_) were determined by averaging all binding curves that matched the theoretical fit with an R^2^ value of 0.96.

### 4.6. Inhibition of SARS-CoV-2 D614G S-Mediated Cell–Cell Fusion

A cell–cell fusion assay was established as previously described [18]. Briefly, effector cells were prepared by transfecting 293T cells with either (1) pcDNA3.1 plasmid co-expressing enhanced green fluorescent protein (EGFP) and the SARS-CoV-2 D614G S protein (designated 293T/EGFP/S), or (2) control plasmid encoding EGFP alone (293T/EGFP). Caco-2 cells were used as target cells. For fusion inhibition evaluation, pre-plated target cells were maintained overnight at 37 °C before co-culturing with effector cell suspensions. Experimental groups received 293T/EGFP/S cells in the presence or absence of test peptides at the indicated concentrations, while control groups received 293T/EGFP cells. Following a 5 h incubation period at 37 °C, fusion inhibition was observed through fluorescence microscopy.

### 4.7. Cell Viability Assay

The cytotoxic effects of peptides on Caco-2 and Calu-6 cells were evaluated using the Cell Counting Kit-8 (Lablead, Beijing, China) following the manufacturer’s protocol. Briefly, target cells were seeded at a density of 10^4^ cells/well in 96-well plates and treated with 100 μL of serially diluted peptides. After 48 h incubation at 37 °C, 10 μL of CCK-8 reagent was added to each well, followed by an additional 2 h incubation at 37 °C. Absorbance at 450 nm was quantified using a microplate reader, and the percentage of cell viability was calculated.

### 4.8. Time-of-Addition Assay

Caco-2 cells plated in 96-well plates were incubated with SARS-CoV-2 pseudovirus, while R1L25HR2 at a final concentration of 1 μM was added 1 h before or 0, 1, 2, 4, 6 or 8 h after the addition of pseudovirus. Following 72 h incubation at 37 °C, cells were lysed and analyzed for pseudovirus entry inhibition efficacy.

### 4.9. Washout Assay

Caco-2 cells plated in 96-well plates were previously incubated with 1 μM R1L25HR2 at 37 °C for 30 min and then washed with DMEM before the addition of SARS-CoV-2 pseudovirus. A normal SARS-CoV-2 pseudovirus inhibition assay with 1 μM R1L25HR2 was also performed as a control.

### 4.10. Attachment Assay

The SARS-CoV-2 pseudovirus and R1L25HR2 were premixed and incubated for 30 min at 37 °C. The Caco-2 cells were plated in 96-well plates and the pseudovirus–inhibitor mixtures were prechilled at 4 °C for 10 min. Then, the cells were incubated with the mixtures at 4 °C for 4 h. After the cells were washed with cold PBS buffer, they were further incubated at 37 °C for 72 h before the determination of luciferase activity as mentioned above.

### 4.11. Statistical Analysis

All data are shown as mean ± SEM. An unpaired non-parametric Mann–Whitney test was performed. * *p* < 0.05 and **** *p* < 0.0001. All statistical analysis was performed using GraphPad Prism (Version 6.02). IC_50_ values were determined by least squares fit nonlinear regression in GraphPad Prism (Version 6.02).

## Figures and Tables

**Table 1:** R1L25HR2’s binding affinity parameters with SARS-CoV-2 RBD and HR1, as determined by BLI.

Protein	K_D_ (M)	k_on_ (1/Ms)	k_off_ (1/s)
RBD	5.02 × 10^−9^	3.81 × 10^4^	1.92 × 10^−4^
HR1	8.52 × 10^−10^	8.88 × 10^4^	7.57 × 10^−5^

## Data Availability

The data presented in this study are available on request from the corresponding author.
